# Screening for Sugarcane Root Phenes Reveals That Reducing Tillering Does Not Lead to an Increased Root Mass Fraction

**DOI:** 10.3389/fpls.2019.00119

**Published:** 2019-02-07

**Authors:** Johann S. Pierre, Jai M. Perroux, Anne L. Rae

**Affiliations:** CSIRO Agriculture and Food, Saint Lucia, QLD, Australia

**Keywords:** biomass allocation, phenotyping, root architecture, root phenes, root systems, *Saccharum spp.*, sugarcane, tiller removal

## Abstract

Sugarcane root systems are poorly studied and understood due to the perennial nature, tall stature, and the long cropping cycle. Whilst some field studies gave insights into sugarcane root traits, there is no detailed description of root and root system traits available. The objectives of our work were to establish a baseline of sugarcane root trait values that will serve for future studies, and to characterize the degree of root system resilience when restricting tiller number. We first conducted an initial screening for root trait diversity on a collection of twenty cultivars representative of sugarcane breeding from 1930 to now. Then we investigated the effect of reduced tillering, via manual de-tillering, on the plant root and root system traits of five varieties grown under optimal conditions in a glasshouse for 1700°Cd. In addition to establishing baseline means and variation for sugarcane root trait values that could serve as a reference for crop models, we demonstrated that the sugarcane root mass fraction was extremely resilient to drastic reduction in tiller number. Restricted plants were effectively maintaining their root system configuration (opening angle) by dramatically increasing the number of nodal roots produced per tiller as well as maximizing total root length by increasing the specific root length. Using this knowledge of sugarcane root traits in combination with the specific agronomic constraints for sugarcane will now underpin the development of a root system ideotype for sugarcane to enable targeted root trait selection for improving crop productivity.

## Introduction

Sugarcane is a tall perennial crop planted vegetatively from setts and allowed to regrow over multiple years after harvest (ratoon crop) (Bonnett, 2013). A fully-developed sugarcane plant consists of multiple tillers (stalks) each having their own root system composed of nodal roots (crown and brace roots) (Smith et al., 2005). Due to the plant’s tall stature (2–3 m) and its perennial nature, the sugarcane root system, in addition to its role in water and nutrient absorption, has an important role to play in the plant’s mechanical anchorage. Desirable ideotypes resist lodging during the cropping season and allow good presentation of the crop for harvesting. The proportion of the root system remaining alive and functional after harvest might also be critical for the performance of the next crop but the fate of sugarcane root system between crop cycles is not clear (Glover, 1968; Ball-Coelho et al., 1992; Smith et al., 2005).

Because of its size and long cropping seasons, between 12 to 15 months for ratoon and planted crops, respectively, it has always been notoriously difficult to study sugarcane root systems in the field on a large scale and with many varieties (Ball-Coelho et al., 1992; Magarey et al., 1999; Chopart et al., 2008; Laclau and Laclau, 2009; Otto et al., 2009). While descriptions of the root system have been derived from trenches in the field (Evans, 1935), we are not aware of any study where sugarcane root and root system architectural traits have been comprehensively assessed in order to find particular root phenotypes that could be used to improve crop yield. So far most, of the analyses of sugarcane root systems have used a limited number of varieties and samples, relying on small soil samples from the field to determine root length density and root biomass and derive information on sugarcane root distribution over time in the soil. From these multiple studies, it has been shown that the maximum rooting depth appears to be between 4.25 m and 4.7 m (Laclau and Laclau, 2009) or even up to 6 m (Evans, 1935) and is little influenced by rainfed or irrigated conditions, which suggests that this trait is under genetic control (Laclau and Laclau, 2009). One study conducted in Brazil reported that 50% of the root biomass was present within the first meter of soil (Laclau and Laclau, 2009), whereas earlier studies conducted in Mauritius and Brazil reported that 80–85% of the roots were present in the top 0.6 m (Blackburn, 1984; Otto et al., 2009) or 65% of the root biomass at harvested concentrated in the top 0.2 m (Otto et al., 2009). The root length density has been reported to be maximum close to the surface, with values ranging from 0.5 to 5.3 cm/cm^3^ (Ball-Coelho et al., 1992; Reghenzani, 1993; Chopart et al., 2008) depending on soil properties, cultivar, and crop age. Given the variability among these observations, it has been noted that bigger scale experiments with larger numbers of varieties and pedoclimatic conditions would be needed to determine a general pattern for sugarcane root biomass distribution.

Phenotyping roots and root architectural traits and understanding their role for crop productivity is currently one of the major challenges for crop improvement (de Dorlodot et al., 2007; Zhu et al., 2011; Meister et al., 2014; Kuijken et al., 2015), with substantial effort in crops like wheat (Wasson et al., 2014; Atkinson et al., 2015) and maize (Trachsel et al., 2011; Wu and Guo, 2014; Zhan et al., 2015). In maize, a list of desired root phenes has been published, known as the ‘steep, deep and cheap’ ideotype for drought and N deficiency (Lynch, 2013). These traits include, for example, abundant root cortical aerenchyma to decrease the metabolic cost of soil exploration, or decreasing the number but increasing the length of each lateral root to maximize resource acquisition and lower root competition. A wheat ideotype has been developed which includes traits such as reducing root length density at the surface to reduce the cost of excessive topsoil exploration, and increasing xylem size to decrease radial resistance to water flow (Wasson et al., 2012). The concepts learned from these root trait ideotypes can also be applied to sugarcane. It is therefore important as a first step for sugarcane improvement to address how diverse sugarcane root and root architectural traits are, and as a second step to identify root phenes that could lead to crop improvement. For example, finding a set of root and root system architectural traits that could improve plant early vigor to give the plant a competitive advantage for soil resource mining will be especially interesting due to the fact that sugarcane is a slow growing crop (Allison et al., 2007) planted during a dry time of the year in a tropical environment highly conducive for fast growing weeds.

The repeat cropping of sugarcane, known as ratooning, places extra demands on the root system compared to an annual crop such as wheat. As in cool climate perennials such as switchgrass, it is likely that below-ground resources which remain in the soil will fuel vigorous regrowth of the following crop. Thus, a larger root system in a perennial crop may have benefits not only for the current season but for subsequent production cycles as well. Increasing the root mass fraction (RMF) (increasing the root:shoot ratio) in wheat has been achieved by restricting tillering, either by genetic selection using near-isogenic lines containing the *tin* gene mutation or by tiller removal (Duggan et al., 2005; Palta et al., 2007; Hendriks et al., 2016). This increase in RMF was due to a greater total length of seminal roots and an increase in root branching in the restricted-tiller plants (Hendriks et al., 2016). In sugarcane, it was shown that root:shoot ratio was quite resilient and returned rapidly to equilibrium in defoliated young plants (Smith, 1998). In both cases, the plant photosynthetic capability was reduced, but in the case of sugarcane, because only the leaves and not the tillers were removed, the entire root system was still present and this could explain the difference in root:shoot ratio behavior between the two crops. A better understanding of the root:shoot ratio resilience in sugarcane would indicate the potential to increase the RMF and contribute to the performance of the next ratoon. Although de-tillering is not feasible as an agronomic treatment, if it successfully increases root system size, it could be used as a screening method to identify varieties with more vigorous root system growth.

In this article, we present the results of a small-pot based screening of sugarcane root traits amongst 20 varieties. We subsequently picked a subset of varieties with contrasted root system phenotypes and, these were investigated further, in large pots, to evaluate the effect of reduced tillering on the plant and its root system phenotype.

Our objectives were to: (i) define root trait phenotypic diversity in sugarcane; and (ii) characterize sugarcane R/S resilience or absence thereof when restricting tiller numbers.

## Materials and Methods

### Experiment 1: Sugarcane Variety Screening for Contrasted Root Traits

#### Plant Materials

Twenty sugarcane varieties were selected based on the dates that they first entered breeding trials (recorded as the year of first planting of seed) in order to obtain a panel of varieties representative of sugarcane breeding from 1930 to now. The sugarcane setts, pieces of stalk with a node including one bud and a ring of root primordia used as planting material, were provided by Sugar Research Australia Ltd. (SRA). To reduce disease susceptibility, setts were treated by immersion in water for 3 h at 50°C and subsequently treated with fungicide (Mancozeb 5 g L^−1^ for 10 min).

#### Growing Conditions

Setts were planted in rectangular trays filled with perlite and incubated at 30°C (12 h day/12 h night) with a light intensity of 500 μmol photon m^−2^ s^−1^. After 8 days or 168°Cd assuming a base temperature of 9°C (Keating et al., 2003), three nodes with sprouted buds of 1–1.5 cm of each variety were selected and planted in 1.5 L round pots filled with UC mix type C (50% sand 50% peat) supplemented with Osmocote (5 g L^−1^). After 27 days (567°Cd), each seedling was re-potted in a 25 L polythene planter bag and plants were transferred to a glasshouse (30°C/13 h, 24°C/11 h) until harvesting at 1060°Cd or 54 days after planting. Plants were manually watered daily.

#### Shoot Measurements and Analysis

At harvest, the number of tillers (emerging shoots + stalks) as well as the number of developed leaves were counted. Developed leaves were defined as leaves where the blade was unfurled so that the dewlap was visible. Leaves and tillers were then pooled together, dried and weighed to obtain the shoot dry weight.

#### Root Measurements and Analysis

Root systems, still attached to the crown, were gently washed and stored in 50% aqueous ethanol at 4°C until scanning. On the day of scanning the root systems were then cut into pieces to be scanned at a resolution of 600 dpi on a flatbed scanner equipped with a transparency unit (Expression 11000XL, Epson, Nagano, Japan) in a shallow A3 tray filled with water. To obtain root descriptors (total root length, root volume, root average diameter, etc.), images were processed with WinRHIZO Pro 2016a software (Regent, Quebec, Canada) using a grayscale thresholding of 200 and filtering out objects with a length:width ratio <4 and objects smaller than 4 cm^2^. After scanning, roots from each plant were dried at 65°C to obtain their dry weight. Using the root dry weight and the Winrhizo data, the specific root length (SRL), defined as the ratio of root length to root dry weight, and the root tissue density (RTD), defined as the ratio of root dry weight to root volume, were calculated. The root mass fraction (RMF) was calculated as the ratio of the root system dry weight to the total plant dry weight.

### Experiment 2: Sugarcane Tillering Experiment

#### Plant Material, Growing Conditions and Harvesting

**FIGURE 1:**
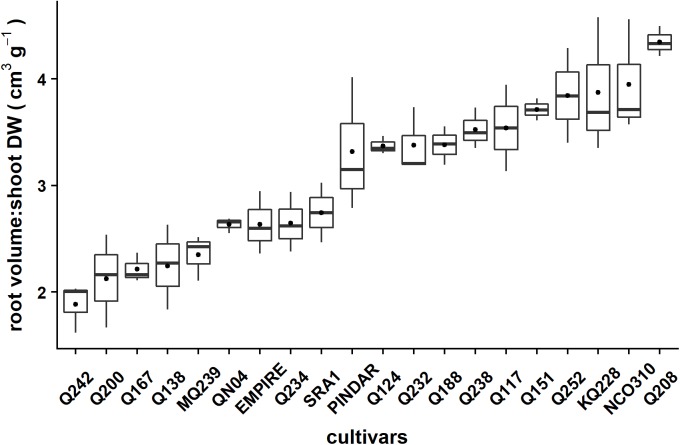
Root volume:shoot DW (cm^3^ g^-1^) for twenty sugarcane varieties. Three plants of each variety were grown in 25 liter planter bags for 1060°Cd (54 days) in a glasshouse (30°C/13 h, 24°C/11 h). In each boxplot, the horizontal black line represents the median and black dot represents the average of the root volume:shoot DW (cm^3^ g^-1^). Varietal differences for the root volume:shoot DW were highly significant (ANOVA; *F*-value = 12.181, *p*-value < 0.001).

Six varieties (Q208, KQ228, Q151, SRA1, MQ239, and Q242) were selected based on contrasting root volume:shoot DW observed in experiment 1 (see Figure 1). Setts were treated in hot water and fungicide as in experiment 1.

#### Growing Conditions

Fifty setts of each variety were planted individually in UC mix type C supplemented with 5 g L^−1^ of Osmocote in small black square pots (5 cm × 8 cm × 14 cm) on the 20^th^ February 2016 and transferred to a glasshouse with natural lighting (30°C for 13 h, 24°C for 11 h). After 21 days or 383°Cd, 12 seedlings of each variety were selected and randomly assigned to one of the two treatments. The first group (*till*+) corresponded to the control group, where no restrictions were imposed on the growing tillers. The second group (*till*−) corresponded to the treatment group, where the number of tillers was restricted to one. Restriction on tillers was imposed by removing any emerging tillers manually each week. Seedlings were planted in tall PVC columns (22.5 cm × 1 m). The internal part of the column was lined with transparent polyethylene film (200 μm thickness), pierced at the bottom to allow free drainage of water. Columns were filled with UC mix type C supplemented with Osmocote (5 g L^−1^). After planting, columns were soaked with water and then subsequently watered via dripping irrigation for 10 min every morning (approx. 2 L daily per pot).

#### Shoot Measurements and Analysis

At harvesting, 94 days after planting (approx. 1700°Cd), the number of stalks and developed leaves were counted. Leaf and stalk were dried separately at 65°C and weighed separately to calculate their dry weight. Total shoot dry weight was calculated as the sum of leaf and stalk mass. Leaf and stalk dry weights were used to calculate the leaf mass fraction (LMF) and stalk mass fraction (SMF) defined as the proportion of the total plant dry weight comprised by the leaves or the stalks, respectively.

#### Root System Washing

The root system with the soil in its sleeve was laid down on the top of the root washing station with a 4.62 mm mesh aperture and washed gently to remove all the soil particles. Root systems, still attached to the crown, were stored in 50% aqueous ethanol at 4°C until further analysis.

#### Root Branching Density

Six 10 cm root segments were cut 30 cm from the base of the crown using 6 randomly selected nodal roots for each plant and the number of lateral roots in each segment was manually counted. The number of lateral roots was then divided by 10 and averaged for each plant to obtain the average branching density per cm of root. The roots were then stored in ethanol as above until being scanned.

#### REST-Root System Architecture

Root systems, still attached to the crown, were cut 30 cm from the crown base. This 30 cm portion of the root system was hung in a black box (106 cm × 106 cm × 106 cm) equipped with two lateral LED lights (Ledgo 150 LED, Ledgo) and images were captured with a tripod-mounted camera (Powershot SX60HS, Canon, Tokyo, Japan). The root system was hung with its largest portion facing the camera. The distance between the root and the camera was fixed and to account for any potential differences a marker of a known size was suspended next to the root system. After imaging, roots were detached from the crown and stored in ethanol as above until being scanned. The crown was dried at 65°C and weighed.

Root system images were then analyzed with REST (Colombi et al., 2015) in semi-automated mode where the user defines the stalk-root interface for every plant. REST selected output variables were the following and are detailed in Supplementary Material (Supplementary Data Sheet 1): (i) root opening angle which corresponds to the opening angle between the left and right outermost angles to the horizontal along an arc of 10 cm; (ii) area of the convex hull that describes the size of the root system in the image; and (iii) the total projected structure length calculated as the sum of the length of root-derived structures and the number of background patches within the convex hull.

#### Root Measurements and Analysis

The number of nodal roots was counted 30 cm from the base of the crown. The root systems were then cut into pieces, scanned, and processed as described for experiment 1. On average, between 25 (*till*−) and 42 (*till*+) images were needed to scan the entire root system. After scanning, roots from each plant were dried at 65°C to obtain their dry weight. Root system dry weight was used to calculate the RMF that represents the proportion of the total weight of the plant comprised by the root system.

#### Statistical Analysis

Data processing, visualization as well as analysis of variance and Tukey *post-hoc* test were done in R (R Core Team, 2017) using the tidyverse (Wickham, 2017) and agricolae (de Mendiburu, 2017) packages.

## Results

### Experiment 1: Sugarcane Root System and Root Trait Diversity in Small Pot Screening

Initially, screening in small pots was carried out to assess the phenotypic diversity of sugarcane RMF, root volume:shoot DW ratios and root structural traits in a collection of commercial varieties representative of sugarcane breeding in Australia from 1930 to the present.

Examining the shoot phenotypes in this experiment, the number of tillers as well as the total number of developed leaves was found to be significantly different among varieties and ranged from 3 to 11.7 for the former and 4 to 7.7 for the latter (Table 1). Similarly, the total root length was highly significantly different among varieties and ranged from 65 to 560 m of root length with an average of 283 m of root length per plant (Table 1). The average root diameter was also significantly different among varieties and ranged from 0.33 to 0.42 mm with an average of 0.37 mm (Table 1). However, some root structural traits did not show variation. SRL (Table 1) as well as RTD (not shown) were not significantly different among varieties and were on average 95.4 m g^−1^ and 5.4 × 10^−2^ g cm^−3^.

**Table 1:** Shoot and root trait variations for twenty sugarcane varieties representative of sugarcane breeding from 1930 to now.

Cultivar	Date	Number of tillers	Number of leaves	Total root length (m)	SRL (cm mg^1^)	Average root diameter (mm)	RMF
EMPIRE	1930	4.3 ± 1.2	6.7 ± 0.6	235.1 ± 27.8	8.48 ± 2.38	0.36 ± 0	0.14 ± 0.04
NCo310	1937	4 ± 1	7 ± 0	168.9 ± 79.3	9.04 ± 1.01	0.37 ± 0.01	0.17 ± 0.02
PINDAR	1937	4 1	4.7 ± 0.6	129.8 ± 109.2	9.03 ± 0.29	0.42 ± 0.01	0.13 ± 0.01
Q117	1963	5.5 ± 0.7	5.5 ± 0.7	272.7 ± 79.3	9.43 ± 0.53	0.38 ± 0.02	0.14 ± 0.02
Q124	1969	5.3 ± 0.6	5.7 ± 0.6	306.3 ± 178.3	Na	0.38 ± 0.02	Na
Q138	1975	6 1	6.7 ± 0.6	239.5 ± 47	9.94 ± 0.6	0.36 ± 0.01	0.11 ± 0.03
Q167	1977	6.7 ± 1.5	5.7 ± 0.6	250.7 ± 92.6	12.07 ± 0.81	0.33 ± 0.02	0.11 ± 0.01
Q151	1981	11.7 ± 2.1	7.3 ± 0.6	421.6 ± 64	10.57 ± 0.12	0.37 ± 0.01	0.15 ± 0.01
Q188	1982	6.3 ± 2.5	5 ± 0	273.7 ± 91.3	9.61 ± 0.79	0.39 ± 0	0.16 ± 0.01
Q208	1987	5.7 ± 0.6	7 ± 0	384.5 ± 120.7	9.49 ± 1.78	0.39 ± 0.04	0.18 ± 0.01
Q234	1988	7.3 ± 0.6	6.3 ± 0.6	345.7 ± 139.2	10.43 ± 2.31	0.37 ± 0.05	0.12 ± 0.01
Q200	1989	6.7 ± 1.5	4 ± 1	194.9 ± 38.3	8.37 ± 0.28	0.41 ± 0.01	0.10 ± 0.02
MQ239	1993	5.7 ± 0.6	7 ± 1	404.5 ± 57.8	11.2 ± 1.73	0.34 ± 0.03	0.12 ± 0.00
Q232	1994	7 ± 1.7	7.3 ± 0.6	455.1 ± 51.3	8.28 ± 2.97	0.37 ± 0.02	0.18 ± 0.07
Q238	1997	6.7 ± 2.1	6.3 ± 0.6	377 ± 217.6	9.53 ± 1.23	0.39 ± 0.02	0.185 ± 0.01
Q242	1997	5.7 ± 1.2	6.7 ± 0.6	132.7 ± 47.2	11.99 ± 2.88	0.36 ± 0.04	0.08 ± 0.01
KQ228	1998	8 ± 4.4	7.7 ± 0.6	560.1 ± 158.8	10.25 ± 4.04	0.36 ± 0.04	0.18 ± 0.04
Q252	2000	5.7 ± 2.9	6 ± 2	244.5 ± 219.5	6.41 ± 2.55	0.41 ± 0.01	0.22 ± 0.07
QN04	2004	3 ± 1	4.3 ± 1.5	65.5 ± 32.1	7.87 ± 1.53	0.41 ± 0.02	0.14 ± 0.04
SRA1	2005	7 ± 0	6.5 ± 0.7	202.8 ± 42.1	8.89 ± 1.14	0.41 ± 0.03	0.11 ± 0.01

*F-value*		*3.174*	*4.902*	*3.565*	*1.598*	*2.971*	*3.736*
p-value		*0.001*	*< 0.001*	*<0.001*	*0.115*	*0.002*	*0.001*

*Three plants of each variety were grown in 25 L planter bags for 1060°Cd (54 days) in a glasshouse (30°C/13 h, 24°C/11 h). Values represent the mean ± standard deviation. Analysis of variance data (at bottom) on the varietal effect for each trait are reported with associated F-values and p-values.*

Variety differences in the relative allocation of biomass to shoot and root were identified. The RMF was highly significantly different among varieties with a minimum of 0.08 for Q242 and a maximum of 0.22 for Q252 and an average of 0.14 (Table 1). Similar results were found when assessing root volume:shoot DW ratio which was highly significant (*p* < 0.001) (Figure 1) among varieties with a minimum of 1.9 cm^3^ g^−1^ for Q242 ranging up to 4.3 cm^3^ g^−1^ for Q208; the average of all varieties was 3.1 cm^3^ g^−1^.

### Experiment 2: Contrasting Phenotypes in Sugarcane Varieties With Restricted Tillering

Based on the initial screening results, six varieties (Q242, MQ239, SRA1, Q151, KQ228, and Q208) were selected for the experiment 2 because of their contrasting root volume:shoot DW. Q208 germinated extremely poorly and only 10 seedlings were replanted into the tall column pots (instead of 12). Their initial size at planting was significantly shorter compared to the seedlings of the other varieties (7.2 cm vs. 42.7 cm on average) and as a result, the growth of the Q208 plants was significantly delayed. This delay had an impact on Q208 final size which artificially increased the strength of the correlations in the following analyses. It was therefore decided to remove Q208 from the analyses. Five varieties (Q242, MQ239, SRA1, Q151, and KQ228) were analyzed for the diversity of shoot traits, root structural traits and root architectural traits both in plants that were allowed to tiller freely and in a treatment where plants were restricted to a single tiller.

#### Above Ground Trait Variation in the Tiller Experiment

**FIGURE 2:**
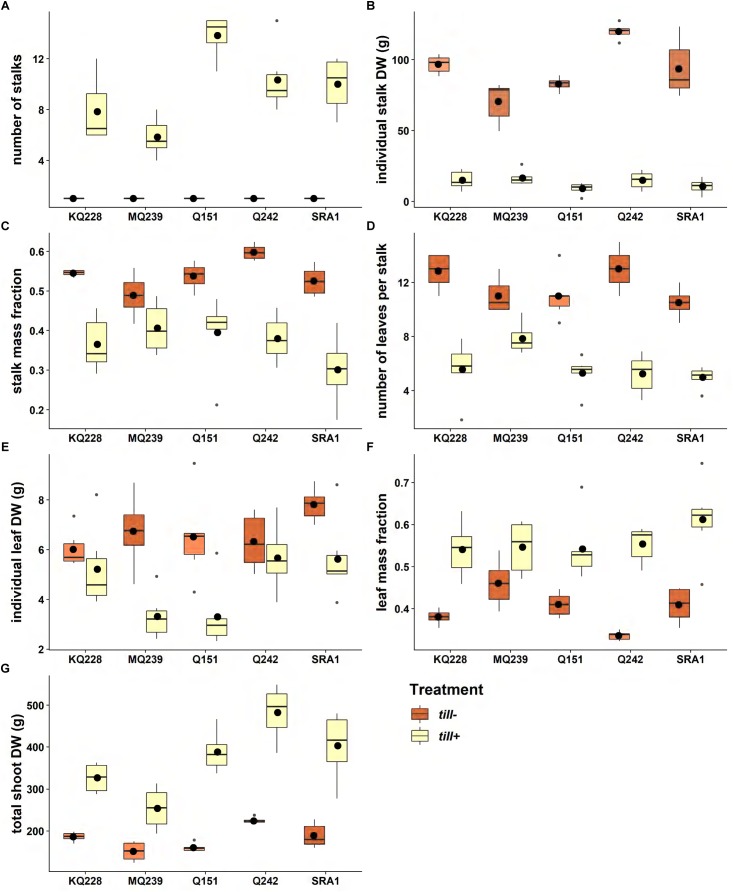
Variation in above ground traits between free (blue) and restricted (red) tillering plants. Six plants of each variety were grown in 40 L tall PVC pots for 1700°Cd (94 days) in a glasshouse (30°C/13 h, 24°C/11 h). In each boxplot, the horizontal line represents the median and the black dot represents the average (*n* = 6). The gray dots represent outliers. The traits measured were **(A)** number of stalks, **(B)** dry weight of individual stalks (g), **(C)** stalk mass fraction, **(D)** number of developed leaves per stalk, **(E)** dry weight of individual leaves (g), **(F)** leaf mass fraction, and **(G)** total shoot dry weight (g). See Supplementary Image 1 for ANOVA and Tukey test tables.

Following the growth of the five contrasting varieties in tall pots, we first measured the phenotypic diversity of the shoot traits and observed how the restriction on tillering affected these traits with a particular emphasis on the SMF and LMF. Within the *till*+ treatment, the average number of tillers ranged from 5.8 for MQ239 to 13.8 for Q151 (Figure 2A) and was significantly different among varieties (*p* < 0.001). The average weight of individual stalks from the *till*+ plants was 13 g and was not significantly different among varieties (Figure 2B). However, the average individual stalk dry weight amongst plants from the *till*− treatment was 85% greater (93 g vs. 13 g) compared to the *till*+ treatment and was highly significantly different (*p* < 0.001) between the treatments (Figure 2B). Furthermore, within the *till*− treatment the individual stalk dry weights were significantly different among varieties (*p* < 0.001) with MQ239 and Q242 at each end of the spectrum (70.5 g vs. 119.9 g) (Figure 2B). Overall, the SMF increased by 31% in the *till*− treatment compared to the *till*+ treatment (Figure 2C).

Consistent with the increase in individual stalk dry weight in the *till*− treatment compared to the *till*+ treatment, differences were also observed for the number of leaves per stalk and the individual leaf dry weights (Figure 2D,E). The number of leaves per stalk increased by 50% from 5.8 for the *till*+ plants to 11.7 for the *till*− plants on average. The dry weight of individual leaves increased by 30% (6.7 g vs. 4.6 g) in the *till*− treatment compared to the *till*+ treatment. While this increase was statistically significant overall when comparing varieties (*p* = 0.007) and treatments (*p* < 0.01), the individual leaf dry weight was not different between treatments for the varieties KQ228 and Q242. Although the single stalks in the *till*− treatment bore larger numbers of leaves per stalk, overall the LMF decreased on average by 24 % in the *till*− treatment compared to the ‘*till*+ treatment (Figure 2F), most likely due to the larger total number of leaf-bearing nodes in the *till*+ plants.

Whilst the individual stalk dry weight, leaf number per stalk and individual leaf dry weight increased in *till*− treatment compared to *till*+ treatment, the overall shoot dry weight was on average 50% higher (*p* < 0.001) in the *till*+ treatment compared to the *till*− treatment (182 g vs. 370 g) (Figure 2G).

#### Root Trait Variation in the Tiller Experiment

Individual root traits were measured to quantify phenotypic changes resulting from the restricted tillering treatment as well the variation due to the differences in genetic background.

**FIGURE 3:**
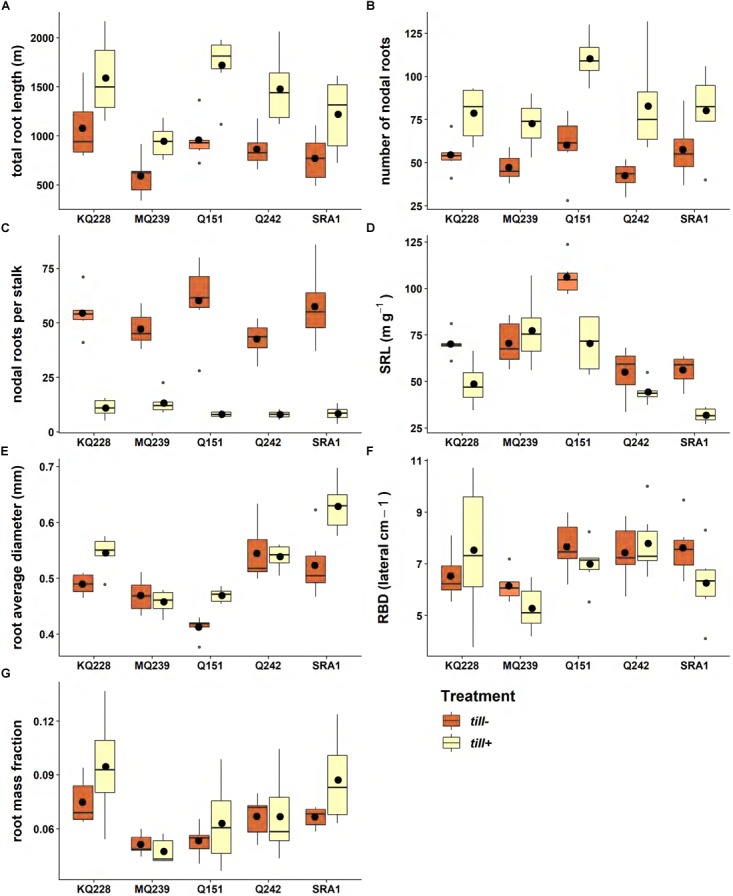
Variations in root traits between free (blue) and restricted (red) tillering plants. Six plants of each variety were grown in 40 L tall PVC pots for 1700°Cd (94 days) in a glasshouse (30°C/13 h, 24°C/11 h). In each boxplot, the horizontal line represents the median and the black dot represents the average (*n* = 6). The gray dots represent outliers. The traits measured were: **(A)** total root length, **(B)** shoot root number, **(C)** nodal roots per stalk, **(D)** SRL, **(E)** root average diameter, **(F)** root branching density, and **(G)** root mass fraction. See Supplementary Image 1 for ANOVA and Tukey test tables.

In the tall pot experiment, the total root length, for *till*+ plants, ranged, on average, from 945 to 1721 m with a significant decrease (*p* < 0.001) in the average total root length of 39% in the *till*− treatment compared to the *till*+ treatment (Figure 3A). This difference was more pronounced for variety Q151, which had a 44% decrease in the root system length, and less pronounced for KQ228 which showed only a 32% reduction.

Total nodal root number was also significantly lower in the restricted tiller treatment (39% lower than in the *till*+ treatment, *p* < 0.001) and no differences were observed among varieties (Figure 3B). Amongst the *till*+ plants, there were few varietal differences and only Q151, with an average total nodal root number of 110, was significantly different from the other varieties. While the total nodal number of roots was higher in the *till*+ treatment, when normalizing this figure to the number of stalks, the number of nodal roots per stalk was 81% higher for the *till*− treatment with an average of 52 nodal roots per stalk (Figure 3C). For the *till*+ treatment, this figure was on average only 10 nodal roots per stalk (Figure 3C).

SRL ranged from 31.9 m g^−1^ to 77.3 m g^−1^ for the *till*+ and was on average, 24% lower than the *till*− treatment (*p* < 0.001) (Figure 3D). Within each treatment significant differences for SRL were observed among varieties; this was particularly noticeable for Q151 in the *till*− treatment, where SRL was significantly different from all the other varieties.

Amongst the *till*+ plants, the root average diameter ranged from 0.46 mm to 0.63 mm with Q151 and SRA1 representing the two extremes (Figure 3E). Overall, the imposed restriction on tillering significantly decreased root diameter by 7% (*p* < 0.001) for *till*− compared to the *till*+ treatment. However, for MQ239 and Q242 the average root diameter was not different between treatments.

The average root branching density (RBD) ranged from 5.3 to 7.8 lateral roots per cm in the *till*+ plants and was not impacted by the restriction on tiller number (Figure 3F). There were small but significant (*p* < 0.01) differences among varieties for RBD.

The RMF highlighted significant differences among varieties (*p* < 0.001) with MQ239 and KQ228 sitting at both ends of the spectrum of the *till*+ plants (Figure 3G). More importantly there was no difference between treatments for this trait, so that within each variety, plants maintained a consistent RMF when tillering was restricted.

#### Root Architecture Variation in the Tiller Experiment

The spatial arrangement of roots is as important for the crop productivity as the individual root traits, and therefore we measured the root system architecture phenotypes as another approach to assess the impact of tillering restriction in the different genetic backgrounds.

**FIGURE 4:**
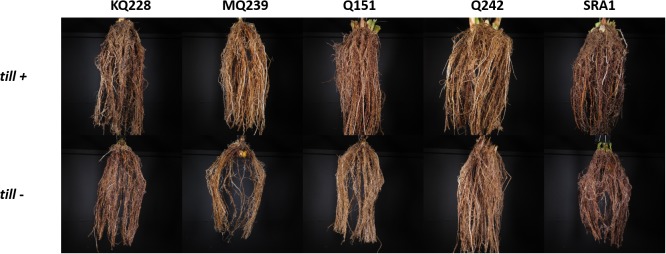
Variations in spatial arrangement of nodal roots *till*+ and *till–* plants. Pictures are a selection of the root system architecture (0 to 30 cm from the crown) of each variety for the two treatments. Root systems were suspended in a black box (106 cm × 106 cm × 106 cm) equipped with two lateral LED lights and images were captured with a tripod-mounted camera.

A selection of representative root system architectures is presented in Figure 4. Whilst some visual differences were apparent for certain traits when comparing between variety and treatment (e.g., root opening angle for Q242 or root system density for MQ239), a simple visual inspection did not reveal any consistent differences among varieties or treatments. Therefore, all the root system images were analyzed with the REST software to measure root system architectural traits.

**FIGURE 5:**
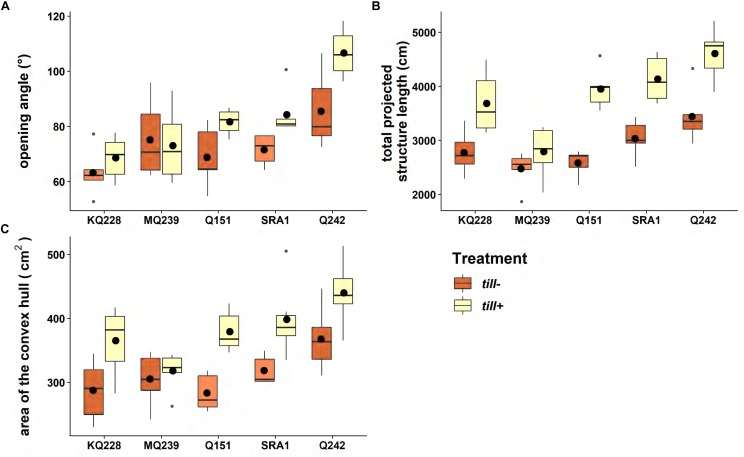
Variations in root system architecture between free (blue) and restricted (red) tillering plants. Six plants of each variety were grown in 40 L tall PVC pots for 1700°Cd (94 days) in a glasshouse (30°C/13 h, 24°C/11 h). In each boxplot, the horizontal line represents the median and the black dot represents the average (*n* = 6). The gray dots represent outliers. The traits measured were (A) root system opening angle (°), (B) total projected structure length (cm), and (C) area of the convex hull (cm^2^). See Supplementary Image 1 for ANOVA and Tukey test tables.

The root system opening angle (Figure 5A) was significantly different among varieties (*p* < 0.001) with KQ228 and Q242 having the narrower and the larger root angles, respectively. Overall, *till*− plants had a significant (*P* < 0.001) decrease in root opening angle. The total projected structure length measured with REST (Figure 5B) was a good trait to discriminate the varietal and treatment effects (*p* < 0.001 for both). Q242 and MQ239 were the two cultivars that had a significantly larger and smaller total projected structure length, respectively. Finally, the area of the convex hull highlighted significant differences for both treatment and variety (*p* < 0.001) but was less discriminant than the total projected structure length for highlighting differences among varieties.

## Discussion

Phenotyping root systems of large crops such as maize or sugarcane has always been challenging both in the field and in controlled environments (Magarey et al., 1999). While digital methods have improved the accuracy compared to previous manual measurements, analysis of whole root systems remains cumbersome and has very low throughput. Nevertheless, data from such studies conducted on a range of cultivars are extremely helpful to provide a baseline and range of variation of root and root system traits. As well as serving as the basis to understand root traits to select for sugarcane improvement, this information can also be used as inputs for physiological crop models. In this study, we established a detailed description of the sugarcane root system and evaluated the response of the root system to shoot biomass restriction.

### Biomass Allocation and Root Mass Fraction

In pot experiment 2, we observed that RMF was not significantly different, for a given cultivar, between the *till*+ and *till*− plants. This tends to agree with the conclusions from two meta-analyses conducted on a large number of species (Poorter and Nagel, 2000a; Poorter et al., 2012b) that demonstrate that, outside of nutrient, temperature and compaction stress, for a given ontogenic stage, biomass allocation patterns tend to be constant. Subsequently, we did not observe any increase in RMF, in contrast to the results demonstrated in wheat (Hendriks et al., 2016). This shows that, in the case of sugarcane, allometric coefficients could not be modified by restrictions imposed on the number of tillers. This is also consistent with previous observations in sugarcane from an experiment where restriction on the above ground biomass was achieved by defoliation but not removal of entire tillers (Smith et al., 2005). Our results now confirm that the difference between wheat, where RMF increased, and sugarcane, where RMF remained constant, was not dependent on the additional roots associated with multiple tillers in sugarcane. This stability of the root DW:shoot DW to pruning has been observed in other grass crops such as barley, where it was demonstrated that the allometric relationship is re-established 7–10 days after removal of 35% of shoot or 48% of roots (Poorter and Nagel, 2000b).

Although, the RMF remained the same, substantial changes were seen in both the shoot and root structure when tillering was restricted. In our experiment, the single stalk of the *till*− plants was seven times heavier than the stalk in *till*+ plants. Such an increase in tiller biomass following tiller removal has been observed in barley (Gu and Marshall, 1988), with a two to fourfold increase in stalk dry weight in glasshouse and field experiments, respectively. This was explained by an increased photosynthetic capacity of the main shoot. In our experiment, in order to maintain the RMF in *till*− plants, the number of nodal roots per stalk increased by 81% and the average root diameter decreased, while SRL increased. This demonstrates that while on a limited carbon budget, the plant will maintain its allometric relationship as well as maximize, as much as possible, its total root length. Similar increases in SRL have been observed in plants on low root carbon budget resulting from nutrient-poor or dry environments (Aerts and Chapin, 1999). In such cases, the increase in SRL is a means for the plant to exploit its available resources as much as possible to mine the soil for nutrients and water. In the present study, without any nutrient or water limitation, the increase in SRL as well as the increase in nodal root number could be more related to anchorage to prevent the lodging of a tall and heavier than normal stalk. In wheat, the lateral spread angle of nodal roots is highly negatively correlated to the lodging rate (Pinthus, 1967), while in maize it was shown that nodal root number is highly correlated to resistance to lodging (Bruce et al., 2001). In our experiment, while the root system size was smaller for the single stalk plants, as shown by the total projected structure length (Figure 5B) and the total root length (Figure 3A), the 81% increase in nodal root number led to root system opening angles that were only 10° narrower on average than the angle in the free tillering plants. This ability to maintain root opening suggests that root system opening angle is highly genetically controlled and that the number of nodal roots is plastic enough to help to maintain this angle.

### SRL in Experiment 2 vs. Previously Published Field Data

Field studies reported mean SRL down to 1 m in the field ranging between 17.6 m g^−1^ to 26.7 m g^−1^ (Magarey et al., 1999; Laclau and Laclau, 2009). Sugarcane crop models use SRL values, derived from field studies, of 5 mg^−1^ in DSSAT (Hoogenboom et al., 2010) or 18 mg^−1^ in APSIM (Keating et al., 2003). In the current study, the average SRL was much higher at 54 m g^−1^ and 71 m g^−1^ for *till*+ and *till*−, respectively. Whilst it is hard to compare the SRL of plants grown in the glasshouse under ideal conditions, to plants grown in the field that could encounter water and nutrient deficit, it seems likely that these differences between field and pot SRL values could be related to the proportion of fine roots which are usually lost and therefore underrepresented in field studies (Roumet et al., 2016). Moreover, water and nutrient limited environments have been shown to favor an increase in SRL (Aerts and Chapin, 1999), hence this tends to suggest that previously published SRL could have been underestimated. In that respect, our sugarcane SRL values, primarily, as well as other root trait values should be tested in crop models to see if they perform better to predict crop growth dynamic and yield. In other crops such as wheat, modeling the effect of specific root parameters on productivity has been a useful approach to identifying beneficial root traits (Lilley and Kirkegaard, 2011).

### Effect of Pot Size on Root Trait Variations

Experiment 1 revealed a range of differences in root trait phenotypes among the twenty varieties. The most relevant differences were around the total root length, the RMF and the root volume:shoot DW. Unlike in wheat where it has been shown that selection has favored a decrease in root:shoot ratio over the last 100 years (Aziz et al., 2017), there was no evidence, in our experiment, that the differences in RMF or root volume:shoot DW were related to the date of origin. These differences were the basis to select cultivars for experiment 2. Whilst an early screening (54 days after planting) for root trait phenotypic differences in small pot is more manageable and allows for the use of a larger panel of cultivars, we found that less variation in root system traits was observed when plants were grown in small pots even though the plants were not root bound. The RMF varied drastically between the two experiments with changes up to 2.7 fold. This could be explained by ontogeny as highlighted in controlled environment experiments in 32 L pots (Smith, 1998). In this experiment, it was demonstrated that sugarcane RMF peaked at 50 days after planting before declining two to threefold until 200 days after planting. Interestingly, the RMF after 125 days in the study by Smith (1998) was between 0.15–0.20 while in our experiment 2 it ranged from 0.07 to 0.1 in the *till*+ conditions. Therefore, this shows that in addition to the ontogeny, using small pots has a negative effect on root system development. Root diameter was also highly different between the two experiments and by far larger and more variable in the experiment 2. Knowing the importance of root diameter on the ability to penetrate compacted soils (Clark et al., 2003) and on water and nutrient uptake (Rieger and Litvin, 1999), this reinforces the idea that large PVC columns like the ones used in our study are more suitable for root phenotyping in controlled environment conditions. Whilst this could seem trivial, a meta-analysis conducted on the results coming from studies where plants were grown in pots has shown that on average, doubling of the pot size increased biomass production by about 43% (Poorter et al., 2012a).

## Conclusion and Future Directions

In this study, using a small set of varieties we have highlighted some interesting contrasted root phenotypes and have established a baseline for sugarcane root trait values for future studies and as input for sugarcane crop models. Furthermore, in the experiment on the effect of tillering restriction on sugarcane root systems, we demonstrated that RMF in each genotype remained unchanged even when tiller number was drastically reduced. The results suggest that it may be difficult overcome the resilience of this trait to increase the allocation of resources and create a larger root system. While the *till*− plants had a smaller root system, they effectively maintained their spatial configuration by greatly increasing the number of nodal roots produced per tiller and by increasing their SRL.

In the future, similar to the work in maize and wheat, we need to develop a root system ideotype for sugarcane focused on the specific agronomic constraints of this crop, characterized for example by high soil compaction and unreliable rainfall during some periods. With this knowledge about an ideal sugarcane root system and our results describing the current structure as a starting point, we would be well positioned to target sugarcane specific root traits for crop improvement.

## Author Contributions

JSP conceived the original project and research plans, carried out the experiments, analyzed the data, and wrote the manuscript. JMP conceived the research plans and carried out the experiments. AR conceived the original project and research plans, wrote, and edited the manuscript.

## Conflict of Interest Statement

The authors declare that the research was conducted in the absence of any commercial or financial relationships that could be construed as a potential conflict of interest.
