# Supplementary material for: Screening for Sugarcane Root Phenes Reveals That Reducing Tillering Does Not Lead to an Increased Root Mass Fraction
Source: Front Plant Sci. 2019 Feb 7;10:119. doi: 10.3389/fpls.2019.00119 (PMC6374332; doi:10.3389/fpls.2019.00119)
Supplement: Supplementary file 1 [file Image_1.PDF]

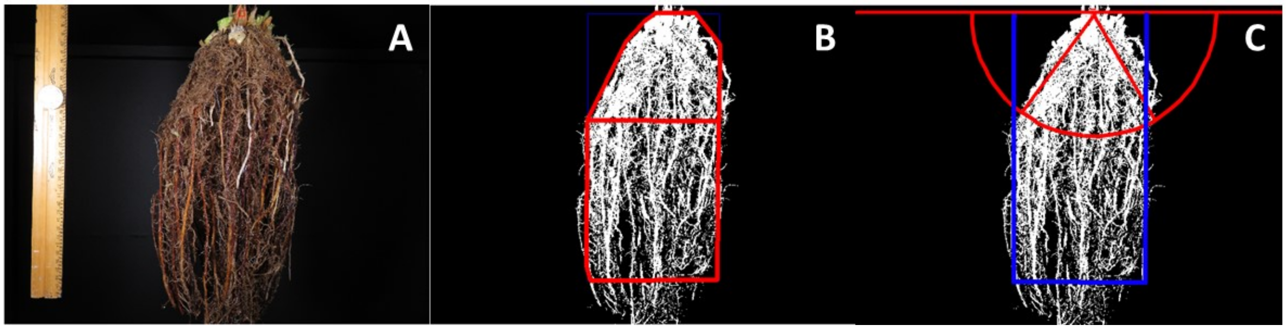


Sup. 1 Root system architecture variables measured by REST software. (A) Original RGB image of the top 30 cm of the root system from the crown base. (B and C) Binary image of the root system. (B) The contour red lines represent the area of the convex hull and the horizontal red line represents the convex hull maximum width. Within the convex hull, the projected total structure length was calculated as the sum of the weighted length of root derived structures and the number of background patches. (C) The blue rectangular contour represents 90% of the region of interest. The horizontal red line represents the soil surface from which a 10 cm (red) arc had been drawn to determine the root opening angle.
